# Neighborhood Socioeconomic Status and Heart Failure or All-Cause Readmission Within a Municipal Hospital System

**DOI:** 10.1016/j.jacadv.2026.103110

**Published:** 2026-08-19

**Authors:** Diane Jeon, Matthew S. Durstenfeld, Yifei Ma, Anjali B. Thakkar, Priscilla Y. Hsue

**Affiliations:** a Division of Cardiology, Department of Medicine, University of California, San Francisco, USA; b Division of Cardiology, Department of Medicine, University of California, Los Angeles, USA

**Keywords:** disease hotspot, heart failure, neighborhood characteristics, patient readmission

## Abstract

**Background:**

Neighborhood socioeconomic status (nSES) is associated with heart failure (HF) outcomes but has not been investigated within a municipal public health system.

**Objectives:**

The authors assessed the relationships between nSES index and HF-specific or all-cause hospital readmissions and all-cause mortality.

**Methods:**

We conducted a retrospective electronic medical record-based cohort study of adults with International Classification of Diseases-9 or -10 diagnosis of HF; geocoded residential address; and index HF admission within a municipal public health system between January 2001 and September 2019. Kaplan-Meier analyses were used for time-to-event analyses. Fine-Gray Survival models were adjusted for demographics, comorbidities, substance use, guideline-directed medical therapy, and 30-day outpatient follow-up with random effects to account for correlation within census tracts.

**Results:**

Among 2,440 individuals, the median age was 60.8 years (SD 14.8 years), 33.6% (821/2,440) were females, and 35.7% (870/2,440) self-identified as Black. The median follow-up time was 38.1 months (IQR: 16.2-74.1 months). The lowest nSES quintile had a 62% higher HF readmission risk (HR: 1.62; 95% CI: 1.09 to 2.41; *P* = 0.018) and 54% higher all-cause readmission risk (HR: 1.54; 95% CI: 1.17-2.02; *P* = 0.002) over 1 year than the highest nSES quintile. nSES was not associated with all-cause mortality. Geographic “hotspots” of index HF hospitalizations or all-cause readmissions occurred in neighborhoods disproportionately affected by historical redlining policies and single-room occupancy units.

**Conclusions:**

Lower nSES is associated with higher HF-specific and all-cause readmission within a municipal hospital system. Future research should focus on improving HF morbidity for readmission “hotspot” neighborhoods.

An estimated 6.7 million American adults live with heart failure (HF).^1^ Individuals with HF have a high risk of hospital readmission and mortality with a median survival of 5 years after diagnosis.^2^ Individual socioeconomic status is an independent predictor for worse HF outcomes, including incidence of HF,^3^ index HF hospitalization,^4^ HF readmission,^5^ and mortality.^6^ Beyond individual socioeconomic status, neighborhood socioeconomic status (nSES) (as well as its inverse neighborhood or social deprivation index) has been associated with HF incidence,^7^^,^^8^ hospitalization or readmission,^9^, ^10^, ^11^ and all-cause mortality.^12^ Thus, even when controlling for individual risk factors, nSES has been associated with worse HF outcomes.

Prior literature including an American Heart Association scientific statement^13^ has highlighted potential mechanisms for how the physical attributes of neighborhoods, such as walkability, access to healthy foods, and air pollution, contribute to the distribution of cardiovascular risk factors such as obesity, diabetes, and hypertension.^14^ Others have hypothesized that social attributes of neighborhoods, including social cohesion, social capital, and crime, may contribute to psychosocial stressors that contribute to cardiovascular risk factors as well as systemic inflammation.^15^ Through different causal pathways, these individual, neighborhood, and systematic factors, including access to health care system and structural racism, may interact to influence individual health outcomes.

Few studies have investigated both HF readmission and all-cause mortality, especially among low-income individuals with public insurance in a single municipal public health system. Moreover, identifying neighborhood “hotspots” of hospital readmissions and mortality can help target programs and policies for the highest-risk communities that would benefit the most from interventions to reduce hospital readmissions and mortality. Although geocoded data have been used to study nSES with HF outcomes,^16^ no prior studies have identified geographic “hotspots” of HF morbidity to our knowledge. In this retrospective cohort study of patients within a single municipal public health system, we aim to: 1) investigate how HF or all-cause readmission, as well as all-cause mortality, differ by nSES; and 2) to identify statistical neighborhood “hotspots” of index HF hospitalization. We hypothesized that higher nSES is associated with decreased HF or all-cause readmission, as well as decreased mortality.

## Methods

### Study population and design

This was a retrospective cohort study of all individuals 18 years or older with a diagnosis of HF designated by International Classification of Diseases (ICD)-9 or 10 code between January 2001 and September 2019 within the San Francisco Health Network, a municipal health system including a public hospital and community clinic network in San Francisco, with an index HF admission and residential address. The diagnosis of HF was defined by ICD-9 code (428, 428.0, 428.1, 428.2X, 428.3X, 428.4, 428.9, 402.01, 402.11, 402.91, 404.01, 404.03, 404.11, 404.13, 404.91, and 404.93) or ICD-10 code (I50.1, I50.20, I50.21, I50.22, I50.23, I50.30, I50.31, I50.32, I50.33, I50.40, I50.41, I50.42, I50.43, I50.9, and I11.0) during an outpatient or inpatient encounter.^17^^,^^18^ Exclusion criteria included the absence of any recorded residential address during the study period or residential address corresponding to Laguna Honda, the largest public skilled nursing facility operated by the San Francisco Department of Public Health which also provides custodial care and rehabilitation services.

### Institutional review board approval

This study was approved by the Institutional Review Board at the University of California, San Francisco, and a waiver of consent was granted. This study was conducted according to the STROBE checklist.

### Outcomes

The primary outcomes are: 1) hospital readmission due to HF or all-causes after index HF hospitalization over 1 year; and 2) all-cause mortality over the follow-up period. Index HF admission is defined as the first hospitalization with primary diagnosis of HF by ICD-9 or ICD-10 code concurrent with or after the first date of HF diagnosis. Follow-up began on the date of index HF hospital admission. HF readmission is defined as any hospitalization with primary diagnosis of HF over 1 year after index HF admission. All-cause readmission is defined as any hospitalization over 1 year after index HF admission. Mortality data were obtained from electronic medical record and the National Death Index during the follow-up period, which ended on September 31, 2019.

### Exposure: neighborhood socioeconomic status

Each patient had 1 residential address assigned from the most recently entered, changed, or verified geocoded address through August 1, 2019. Residential addresses were previously extracted on June 1, 2021, and geocoded to latitude and longitude coordinates (UCSF Population Health Data Initiative) which were then assigned to census tracts according to 2010 U.S. Census data in ArcGIS Pro (ESRI 2020). Neighborhood SES index is a composite measure of 7 indicators including income, poverty, housing cost, rental cost, education, occupation, and employment incorporating 2013 to 2017 American Community Survey data and previously developed using principal components analysis based on the established nSES index.^19^, ^20^, ^21^ Neighborhood socioeconomic index was analyzed by quintiles based on distribution of nSES index scores for all census tracts in the 9-county San Francisco Bay area from quintile 1 (lowest nSES) to quintile 5 (highest nSES).

### Covariates

Demographic and clinical data at the time of HF diagnosis were extracted from the electronic health record. Comorbidities were identified by ICD-9 or ICD-10 code with a 1-year lookback before the diagnosis of HF. Substance use was defined by positive urine toxicology or ICD-9 or ICD-10 code. Echocardiograms were concurrent with or after HF diagnosis and were extracted as structured text from clinical reports. HF with reduced ejection fraction (HFrEF) was defined as left ventricular ejection fraction < 40% or qualitative assessment as left ventricle ejection fraction was not commonly reported in earlier study years. Medication prescriptions were identified for the subset of participants with an index HF admission based on admission medication reconciliation and discharge prescriptions. Medical therapy for HF was defined as prescription of any guideline-directed medical therapy medication during the study period (including beta-blocker, angiotensin-converting enzyme inhibitor, angiotensin II receptor blocker, angiotensin receptor/neprilysin inhibitor as well as mineralocorticoid antagonist).

### Mapping

Using ArcGIS, geocoded coordinates of all individuals with either hospitalization and readmission due to HF or all-cause mortality during the follow-up period were mapped. Relative density and statistical hotspots were mapped in ArcGIS, the latter using the hotspot analysis tool which calculates the Getis-Ord Gi∗ statistic (ESRI). Redlining maps were used with permission from published data (University of Richmond).

### Statistical methods

Baseline demographics across nSES quintiles were described using the Kruskal-Wallis test or chi-square test. Kaplan-Meier curves were used to plot time-to-event analyses for both readmission and mortality. Fine-Gray survival models were used in multivariable analyses to examine the association between nSES and readmission that account for competing risks of death before readmissions with random effects to account for correlation within census tracts. We formally tested for the proportional hazard assumption by interacting nSES with log-survival time in all the survival models, and *P* value >0.05 indicated no violations. Multivariable models were adjusted for selected covariates including demographics (age, sex, and self-reported race/ethnicity), comorbidities (hypertension, diabetes, chronic kidney disease, and atrial fibrillation), HFrEF, substance use (alcohol, tobacco, methamphetamine, or cocaine use), guideline-directed medical therapy (any prescription of any medication including beta-blocker, angiotensin-converting enzyme inhibitor, angiotensin II receptor blocker, and angiotensin receptor/neprilysin inhibitor, mineralocorticoid antagonist or loop diuretic during the study period), outpatient follow-up within 30 days of hospital discharge, and insurance type. A 2-tailed *P* value <0.05 was considered statistically significant. All statistical analyses were conducted in SAS (version 9.4M7; SAS Institute).

## Results

### Baseline characteristics

**Figure 1:**
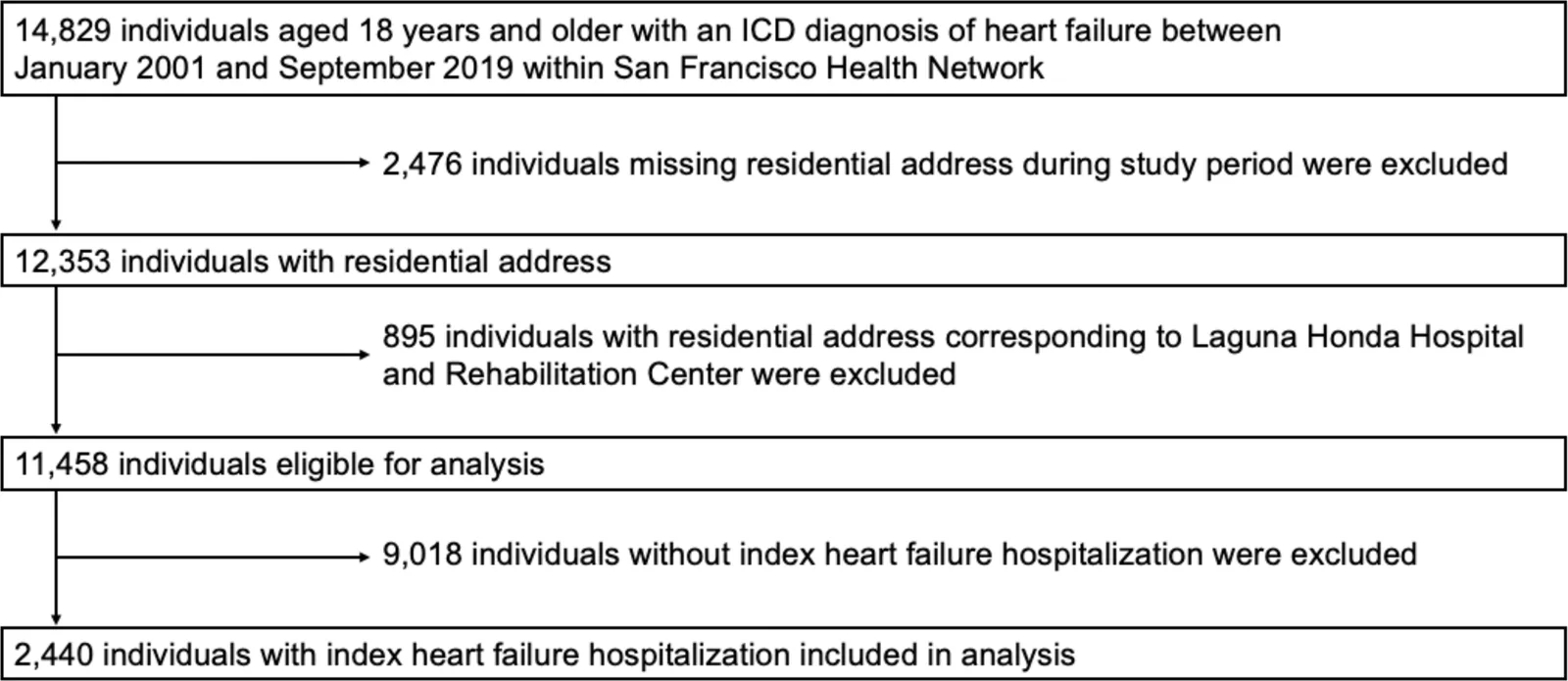
**Flowsheet of Study Cohort** ICD = International Classification of Disease.

Of the 14,829 individuals with a diagnosis of HF between January 2001 and September 2019 at a municipal health system in San Francisco, 11,458 individuals had a residential address, of whom 2,440 individuals (21%) had an index HF admission (Figure 1). Compared with individuals without index HF admission (n = 9,018), individuals with an index HF admission (n = 2,440) were more likely to be male and have Medicaid. They were also more likely to have comorbidities including coronary artery disease, HFrEF, and hypertension as well as documented substance use (Table 1).

**Table 1:** Baseline Characteristics for Individuals Without Index Heart Failure Admission Compared With Individuals With Index Heart Failure Admission

	Participants without Index HF Admission (n = 9,018)	Participants with Index HF Admission (n = 2,440)	Total (N = 11,458)	*P* Value
Mean age at time of index admission, y, mean ± SD	N/A	60.8 ± 14.8	N/A	
Female	3,856 (42.8%)	821 (33.6%)	4,677 (40.8%)	<0.0001
Self-identified race				
White	2,476 (27.5%)	500 (20.5%)	2,976 (26.0%)	<0.0001
Black	2,247 (24.9%)	870 (35.7%)	3,117 (27.2%)	
Hispanic	1,075 (11.9%)	288 (11.8%)	1,363 (11.9%)	
Asian/Pacific Islander	2,198 (24.4%)	530 (21.7%)	2,728 (23.8%)	
Other	1,022 (11.3%)	252 (10.3%)	1,274 (11.1%)	
Insurance status				
Medicare	3,757 (41.7%)	1,045 (42.8%)	4,802 (41.9%)	<0.0001
Medicaid	2,350 (26.1%)	914 (37.5%)	3,264 (28.5%)	
Commercial	127 (1.4%)	18 (0.7%)	145 (1.3%)	
Uninsured	319 (3.5%)	89 (3.6%)	408 (3.6%)	
Other	2,465 (27.3%)	374 (15.3%)	2,839 (24.8%)	
Comorbidities				
Atrial fibrillation	2,356 (26.1%)	670 (27.5%)	3,026 (26.4%)	0.18
Coronary artery disease	2,157 (23.9%)	655 (26.8%)	2,812 (24.5%)	0.0029
Chronic kidney disease	1,378 (15.3%)	330 (13.5%)	1708 (14.9%)	0.031
Diabetes mellitus	874 (9.7%)	212 (8.7%)	1,086 (9.5%)	0.13
Heart failure with reduced LVEF <40%	1,693 (25.0%), n = 6,775	1,201 (51.8%), n = 2,317	2,894 (31.8%), n = 9,092	<0.0001
Hypertension	7,273 (80.6%)	2,097 (85.9%)	9,370 (81.8%)	<0.0001
Prior myocardial infarction	1,294 (14.3%)	407 (16.7%)	1,701 (14.8%)	0.0041
Substance use^a^				
Alcohol	1,704 (18.9%)	725 (29.7%)	2,429 (21.2%)	<0.0001
Tobacco	3,430 (38.0%)	1,503 (61.6%)	4,933 (43.1%)	<0.0001
Cannabis	407 (4.5%)	216 (8.9%)	623 (5.4%)	<0.0001
Methamphetamine	919 (10.2%)	540 (22.1%)	1,459 (12.7%)	<0.0001
Cocaine	1,022 (11.3%)	608 (24.9%)	1,630 (14.2%)	<0.0001
Opioid	899 (10.0%)	337 (13.8%)	1,236 (10.8%)	<0.0001

Baseline characteristics at the time of diagnosis of heart failure unless otherwise noted. *P* values were estimated using the chi-square test.

HF = heart failure; LVEF = left ventricular ejection fraction; N/A = not applicable.

a Based on ICD-9 or ICD-10 code and urine toxicology screen.

Among the study cohort (*n* = 2,440), the median age was 60.8 years (SD 14.8 years) at the time of index HF admission, 33.6% (821/2,440) were females, and 35.7% (870/2,440) identified as Black (Table 2). Covariates including age, self-identified race, insurance type, and substance use differed across nSES quintiles (Table 2). However, the percentage of HFrEF did not differ across nSES quintiles. Individuals in the lowest nSES quintile 1 were more likely to be younger, Black, have Medicaid, and have documented substance use. Prescription of guideline-directed medical therapy and 30-day outpatient follow-up did not differ significantly across nSES quintiles.

**Table 2:** Baseline Characteristics for Individuals With Index Heart Failure Admission for nSES Quintiles 1 (Lowest) Through 5 (Highest)

	nSES Quintile 1 (Lowest) (n = 1,006)	nSES Quintile 2 (n = 638)	nSES Quintile 3 (n = 323)	nSES Quintile 4 (n = 308)	nSES Quintile 5 (Highest) (n = 165)	Total (N = 2,440)	*P* Value
Mean age at time of index admission, y, mean ± SD	59.8 ± 14.3	61.3 ± 15.7	62.3 ± 15.2	61 ± 14.5	61.3 ± 14.2	60.8 ± 14.8	0.085
Female	340 (33.8%)	217 (34.0%)	120 (37.2%)	100 (32.5%)	44 (26.7%)	821 (33.6%)	0.23
Self-identified race							
White	138 (13.7%)	125 (19.6%)	74 (22.9%)	90 (29.2%)	73 (44.2%)	500 (20.5%)	<0.0001
Black	480 (47.7%)	192 (30.1%)	69 (21.4%)	90 (29.2%)	39 (23.6%)	870 (35.7%)	
Hispanic	102 (10.1%)	86 (13.5%)	61 (18.9%)	30 (9.7%)	9 (5.5%)	288 (11.8%)	
Asian/Pacific Islander	207 (20.6%)	150 (23.5%)	81 (25.1%)	67 (21.8%)	25 (15.2%)	530 (21.7%)	
Other	79 (7.9%)	85 (13.3%)	38 (11.8%)	31 (10.1%)	19 (11.5%)	252 (10.3%)	
Insurance status							
Medicare	433 (43.0%)	263 (41.2%)	132 (40.9%)	147 (47.7%)	70 (42.4%)	1,045 (42.8%)	0.048
Medicaid	398 (39.6%)	248 (38.9%)	111 (34.4%)	96 (31.2%)	61 (37.0%)	914 (37.5%)	
Commercial	6 (0.6%)	4 (0.6%)	5 (1.5%)	0 (0.0%)	3 (1.8%)	18 (0.7%)	
Uninsured	30 (3.0%)	28 (4.4%)	16 (5.0%)	8 (2.6%)	7 (4.2%)	89 (3.6%)	
Other	139 (13.8%)	95 (14.9%)	59 (18.3%)	57 (18.5%)	24 (14.5%)	374 (15.3%)	
Comorbidities							
Atrial fibrillation	255 (25.3%)	175 (27.4%)	101 (31.3%)	89 (28.9%)	50 (30.3%)	670 (27.5%)	0.23
Coronary artery disease	244 (24.3%)	185 (29.0%)	88 (27.2%)	86 (27.9%)	52 (31.5%)	655 (26.8%)	0.14
Chronic kidney disease	140 (13.9%)	82 (12.9%)	47 (14.6%)	41 (13.3%)	20 (12.1%)	330 (13.5%)	0.92
Diabetes mellitus	90 (8.9%)	57 (8.9%)	28 (8.7%)	28 (9.1%)	9 (5.5%)	212 (8.7%)	0.67
Heart failure with reduced LVEF <40%	472 (49.4%), n = 955	328 (54.3%), n = 604	150 (48.9%), n = 307	163 (55.4%) n = 294	88 (56.1%), n = 157	1,201 (51.8%), n = 2,317	0.11
Hypertension	872 (86.7%)	556 (87.1%)	283 (87.6%)	257 (83.4%)	129 (78.2%)	2,097 (85.9%)	0.019
Prior myocardial infarction	168 (16.7%)	125 (19.6%)	41 (12.7%)	46 (14.9%)	27 (16.4%)	407 (16.7%)	0.082
Substance use^a^							
Alcohol	326 (32.4%)	192 (30.1%)	74 (22.9%)	78 (25.3%)	55 (33.3%)	725 (29.7%)	0.0057
Tobacco	656 (65.2%)	399 (62.5%)	175 (54.2%)	172 (55.8%)	101 (61.2%)	1,503 (61.6%)	0.0015
Cannabis	95 (9.4%)	75 (11.8%)	19 (5.9%)	24 (7.8%)	3 (1.8%)	216 (8.9%)	0.0003
Methamphetamine	247 (24.6%)	153 (24.0%)	48 (14.9%)	59 (19.2%)	33 (20.0%)	540 (22.1%)	0.0023
Cocaine	295 (29.3%)	160 (25.1%)	57 (17.6%)	59 (19.2%)	37 (22.4%)	608 (24.9%)	<0.0001
Opioids	165 (16.4%)	93 (14.6%)	26 (8.0%)	33 (10.7%)	20 (12.1%)	337 (13.8%)	0.0013
Medication prescription^b^							
Beta-blockers	438 (43.5%)	300 (47.0%)	146 (45.2%)	142 (46.1%)	71 (43.0%)	1,097 (45.0%)	0.67
Angiotensin-converting enzyme inhibitors	443 (44.0%)	302 (47.3%)	133 (41.2%)	137 (44.5%)	74 (44.8%)	1,089 (44.6%)	0.46
Angiotensin receptor blockers	121 (12.0%)	82 (12.9%)	37 (11.5%)	42 (13.6%)	18 (10.9%)	300 (12.3%)	0.87
Angiotensin receptor-neprilysin inhibitor	16 (1.6%)	14 (2.2%)	9 (2.8%)	5 (1.6%)	4 (2.4%)	48 (2.0%)	0.66
Mineralocorticoid receptor antagonist	203 (20.2%)	127 (19.9%)	67 (20.7%)	51 (16.6%)	39 (23.6%)	487 (20.0%)	0.44
Loop diuretic	469 (46.6%)	312 (48.9%)	155 (48.0%)	145 (47.1%)	74 (44.8%)	1,155 (47.3%)	0.86
Outpatient 30-d follow-up	704 (70.0%)	433 (67.9%)	235 (72.8%)	228 (74.0%)	115 (69.7%)	1,715 (70.3%)	0.3

Baseline characteristics at the time of diagnosis of heart failure unless otherwise noted. *P* values were estimated using the chi-square test except for the covariates age and first ejection fraction after heart failure diagnosis for which the Kruskal-Wallis test was used.

nSES = neighborhood socioeconomic status; other abbreviations as in Table 1.

a Based on ICD-9 or ICD-10 code and urine toxicology screen.

b Prescription of any guideline-directed therapy medication during study period.

### Hospital readmission after index HF hospitalization over 1 year

**Figure 2:**
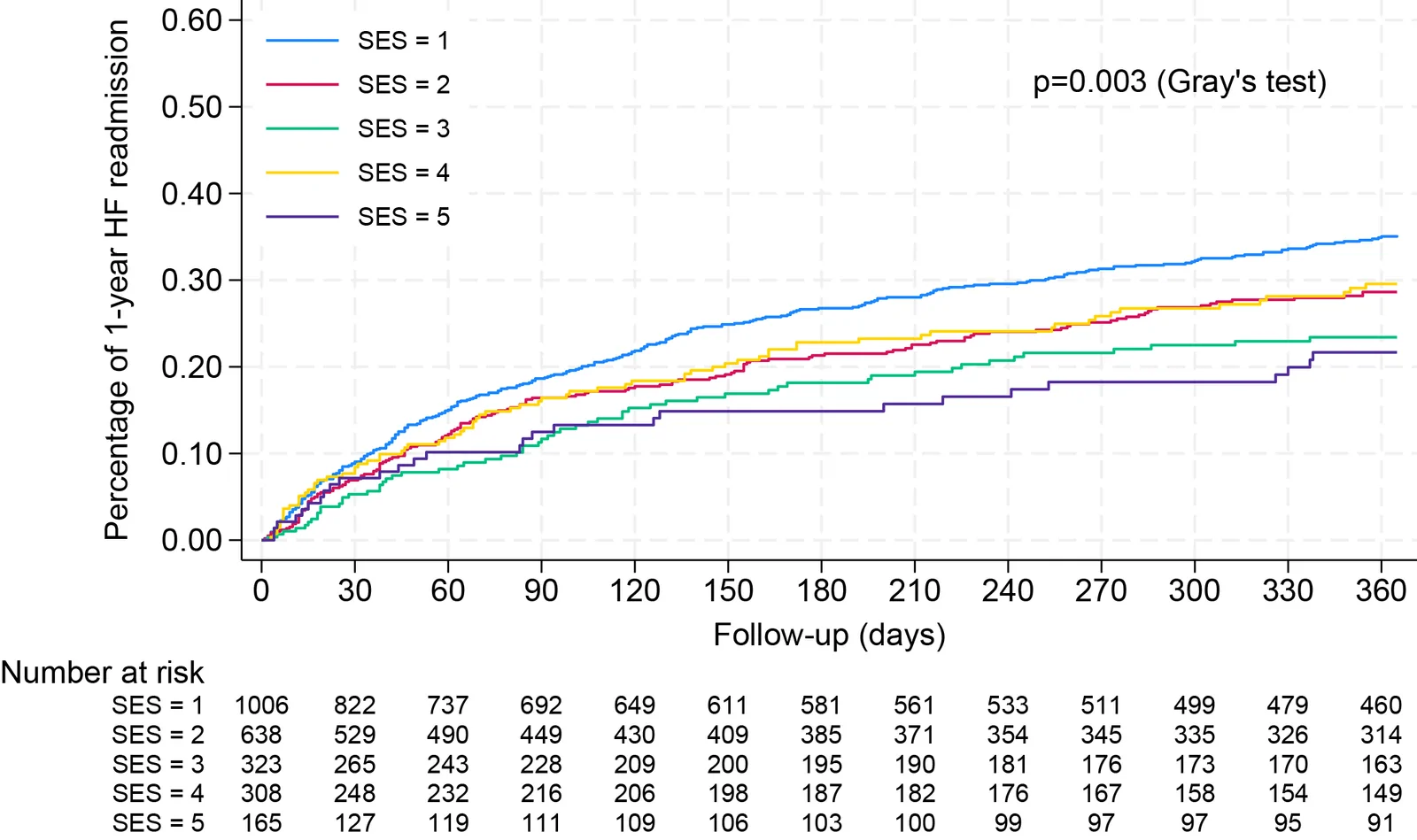
**Cumulative First Heart Failure Readmission Over 1 Year After Index Heart Failure Hospitalization by Neighborhood Socioeconomic Status Quintile** Unadjusted cumulative 1-year heart failure readmission for participants by neighborhood socioeconomic status quintile. At-risk tables are shown at the bottom. HF = heart failure; SES = neighorhood socioeconomic status.

**Figure 3:**
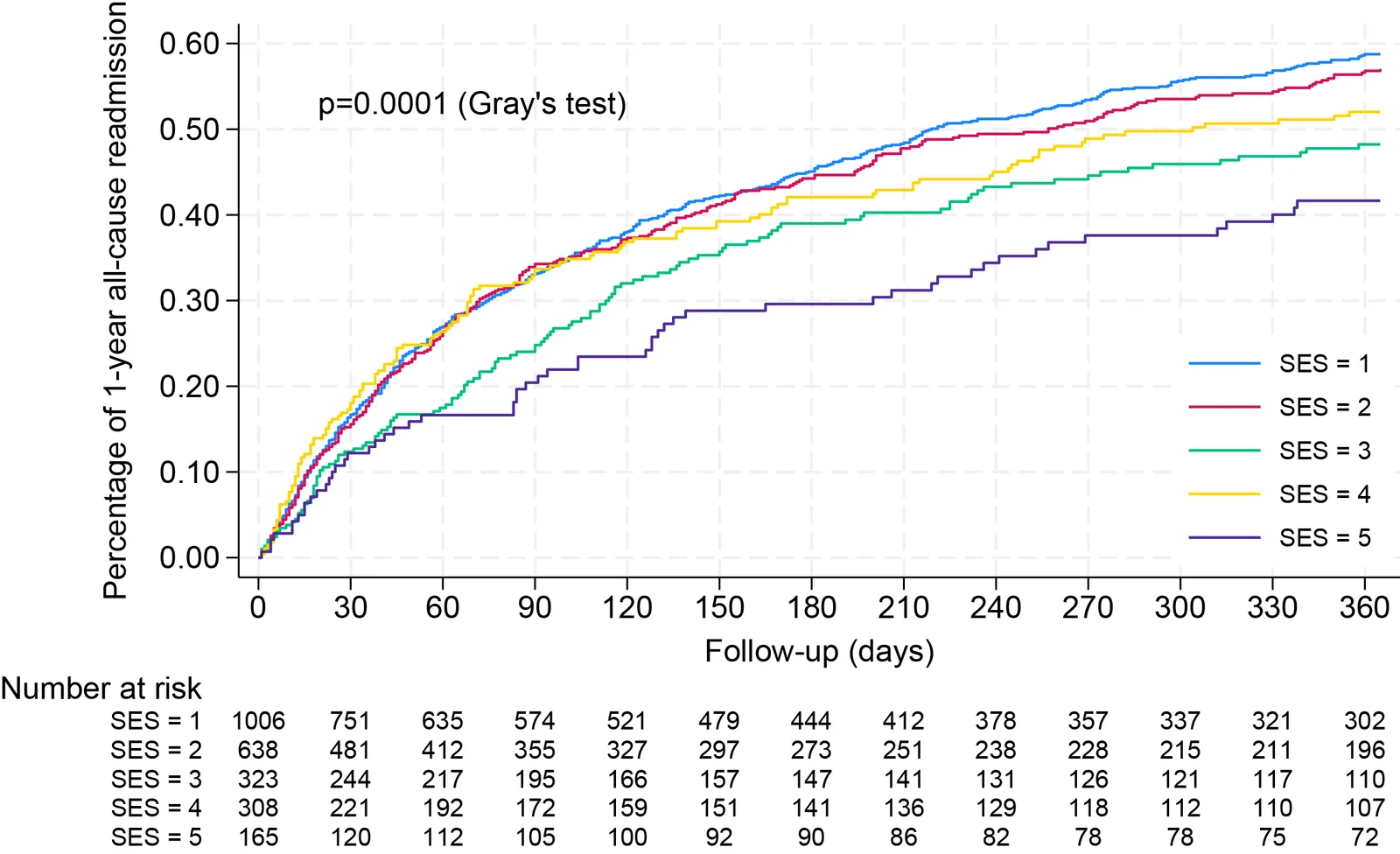
**Cumulative First All-Cause Readmission Over 1 Year After Index Heart Failure Hospitalization by Neighborhood Socioeconomic Status Quintile** Unadjusted cumulative 1-year all-cause readmission for participants by neighborhood socioeconomic status quintile. At-risk tables are shown at the bottom. Abbreviation as in Figure 2.

The median follow-up period was 38.6 months (IQR: 16.8-76.1 months) for HF readmission and 38.1 months (IQR: 16.2-74.1 months) for all-cause readmission. In unadjusted analyses for 2,440 individuals with geocoded data, a diagnosis of HF, and index HF hospitalization, 617 individuals (25.3%) were readmitted due to HF and 1,137 individuals (46.6%) were readmitted due to any cause within 1 year after index HF hospitalization. In unadjusted chi-square analyses, nSES is associated with cumulative HF readmission over 1 year (*P* = 0.007) and cumulative all-cause readmission over 1 year (*P* = 0.0002) after index HF admission. In unadjusted time-to-event analyses, nSES is associated with a risk of HF readmission over 1 year (*P* = 0.003) (Figure 2) and all-cause readmission over 1 year (*P* = 0.0001) (Figure 3).

In multivariable analyses, the lowest nSES quintile 1 had a 62% higher HF readmission risk over 1 year (HR: 1.62; 95% CI: 1.09-2.41; *P* = 0.018) and 54% higher all-cause readmission risk (HR: 1.54; 95% CI: 1.17-2.02; *P* = 0.002) compared with the highest nSES quintile 5 (Table 3). Including guideline-directed medical therapy, 30-day outpatient follow-up, and insurance type in multivariable analyses does not significantly change these findings (Supplemental Table 1).

**Table 3:** Multivariable Model for Heart Failure or All-Cause Readmission Over 1 Year After Index Heart Failure Hospitalization

nSES Quintile	Heart Failure Readmission Over 1 Year^a^	All-Cause Readmission Over 1 Year^b^
	Subdistribution HR (95% CI)	
nSES quintile 1 (lowest)	1.62 (1.09, 2.41), *P* = 0.018	1.54 (1.17, 2.02), *P* = 0.002
nSES quintile 2	1.35 (0.9, 2.04), *P* = 0.15	1.54 (1.16, 2.04), *P* = 0.003
nSES quintile 3	1.08 (0.68, 1.7), *P* = 0.745	1.17 (0.86, 1.61), *P* = 0.315
nSES quintile 4	1.52 (0.98, 2.34), *P* = 0.061	1.54 (1.14, 2.09), *P* = 0.006
nSES quintile 5 (highest)	Reference	Reference

Multivariable analyses of the association between nSES quintile and HF or all-cause readmission over 1 year after index HF hospitalization adjusted for demographics (age, sex, and self-reported race), comorbidities (hypertension, diabetes, chronic kidney disease, and atrial fibrillation), type of HF (HFrEF), and substance use (alcohol, tobacco, methamphetamine, or cocaine). HRs compare each nSES quintile with the highest nSES quintile, 5.

Abbreviation as in Table 2.

a Adjusted HR for heart failure readmission over 1-year after index HF hospitalization: 0.92 (95% CI: 0.86-0.99), *P* trend = 0.027.

b Adjusted HR for all-cause readmission over 1-year after index HF hospitalization: 0.94 (95% CI: 0.90-0.99), *P* trend = 0.015.

### All-cause mortality

**Figure 4:**
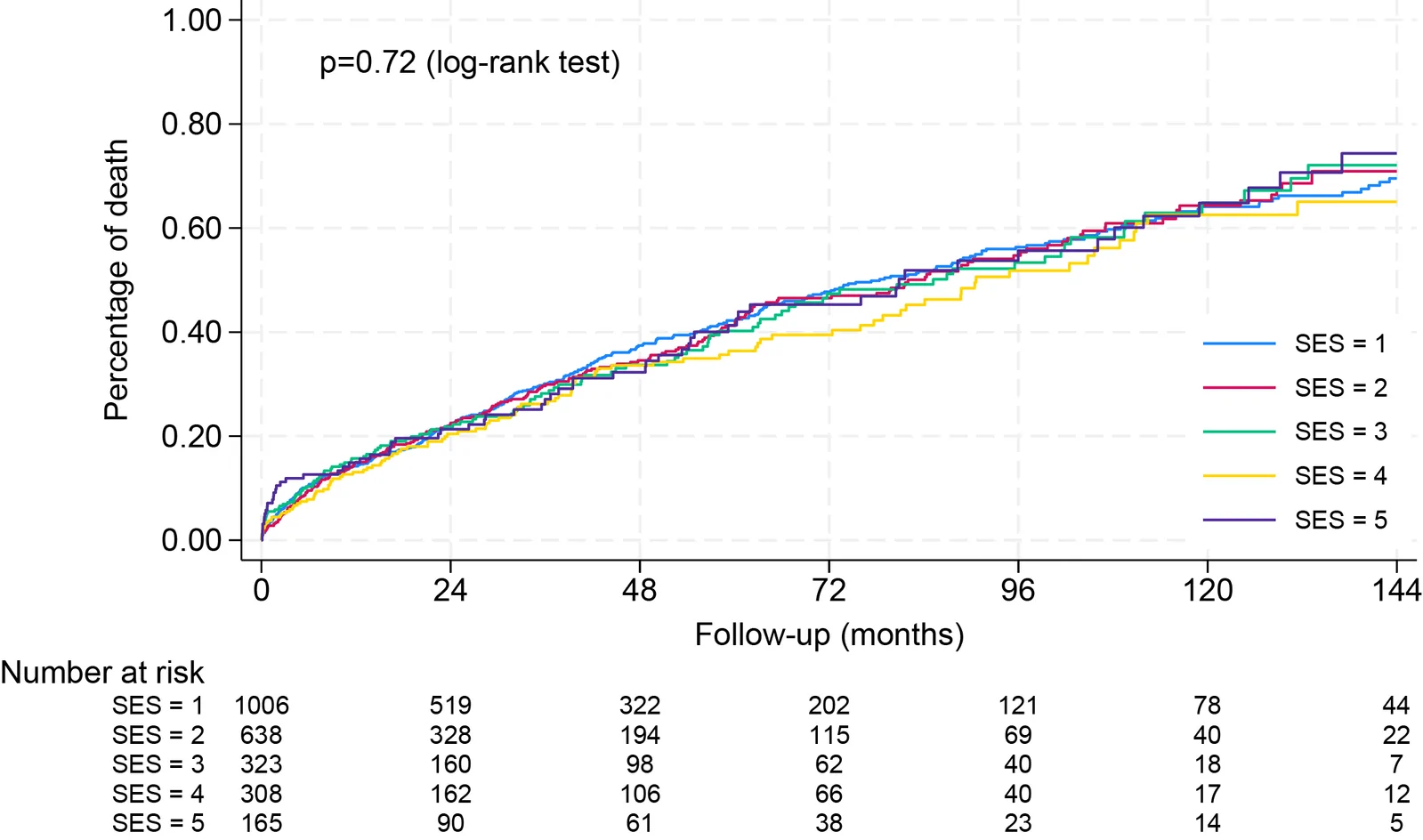
**Kaplan-Meier Curves for Mortality by Neighborhood Socioeconomic Status Quintile** Unadjusted cumulative mortality by neighborhood socioeconomic status quintile. At-risk tables are shown at the bottom. Abbreviation as in Figure 2.

In unadjusted time-to-event analyses, nSES is not associated with all-cause mortality over the follow-up period (*P* = 0.72) (Figure 4). Adjusted for demographics, comorbidities, and substance use, nSES is not associated with all-cause mortality (HR: 0.99 per 1 quintile change in nSES; 95% CI: 0.69-1.20; *P* = 0.509) (Table 4). Including guideline-directed medical therapy, 30-day outpatient follow-up and insurance type in multivariable analyses does not significantly change these findings (Supplemental Table 2).

**Table 4:** Multivariable Model for All-Cause Mortality

nSES Quintile	Survival Over Follow-Up Period^a^ HR (95% CI)
nSES quintile 1 (lowest)	0.91 (0.69, 1.20), *P* = 0.509
nSES quintile 2	0.92 (0.69, 1.22), *P* = 0.554
nSES quintile 3	0.88 (0.64, 1.20), *P* = 0.412
nSES quintile 4	0.80 (0.59, 1.10), *P* = 0.167
nSES quintile 5 (highest)	Reference

Multivariable analyses of the association between nSES quintile and all-cause mortality over study period adjusted for demographics (age, sex, and self-reported race), comorbidities (hypertension, diabetes, chronic kidney disease, and atrial fibrillation), type of HF (HFrEF), and substance use (alcohol, tobacco, methamphetamine, or cocaine). HRs compare each nSES quintile with the highest nSES quintile, 5.

Abbreviation as in Table 2.

a Adjusted HR for all-cause mortality: 0.99 (95% CI: 0.94-1.04), *P* trend = 0.706.

### Mapping

**Figure 5:**
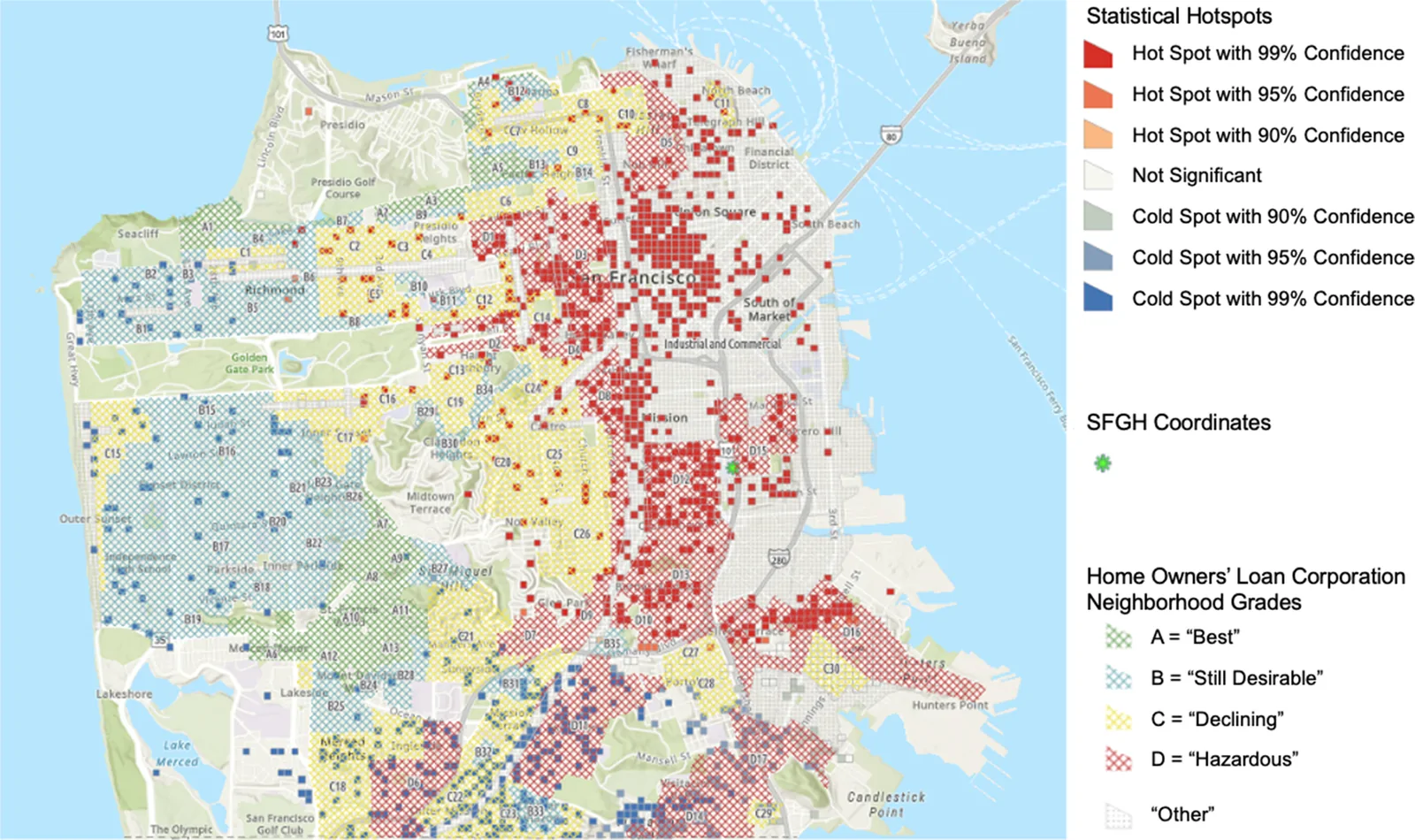
**Statistical Hot Spots of Individuals with Index Heart Failure Hospitalization and Historical Redlining** Statistically significant hotspots of index HF hospitalizations overlaid with neighborhood grading by Home Owners’ Loan Corporation established in 1933. Most statistical hotspots of index HF hospitalizations occur in the lowest grade of neighborhood, “D” (hazardous), and second-lowest grade of neighborhood, “C” (declining). SFGH = San Francisco General Hospital.

**Figure 6:**
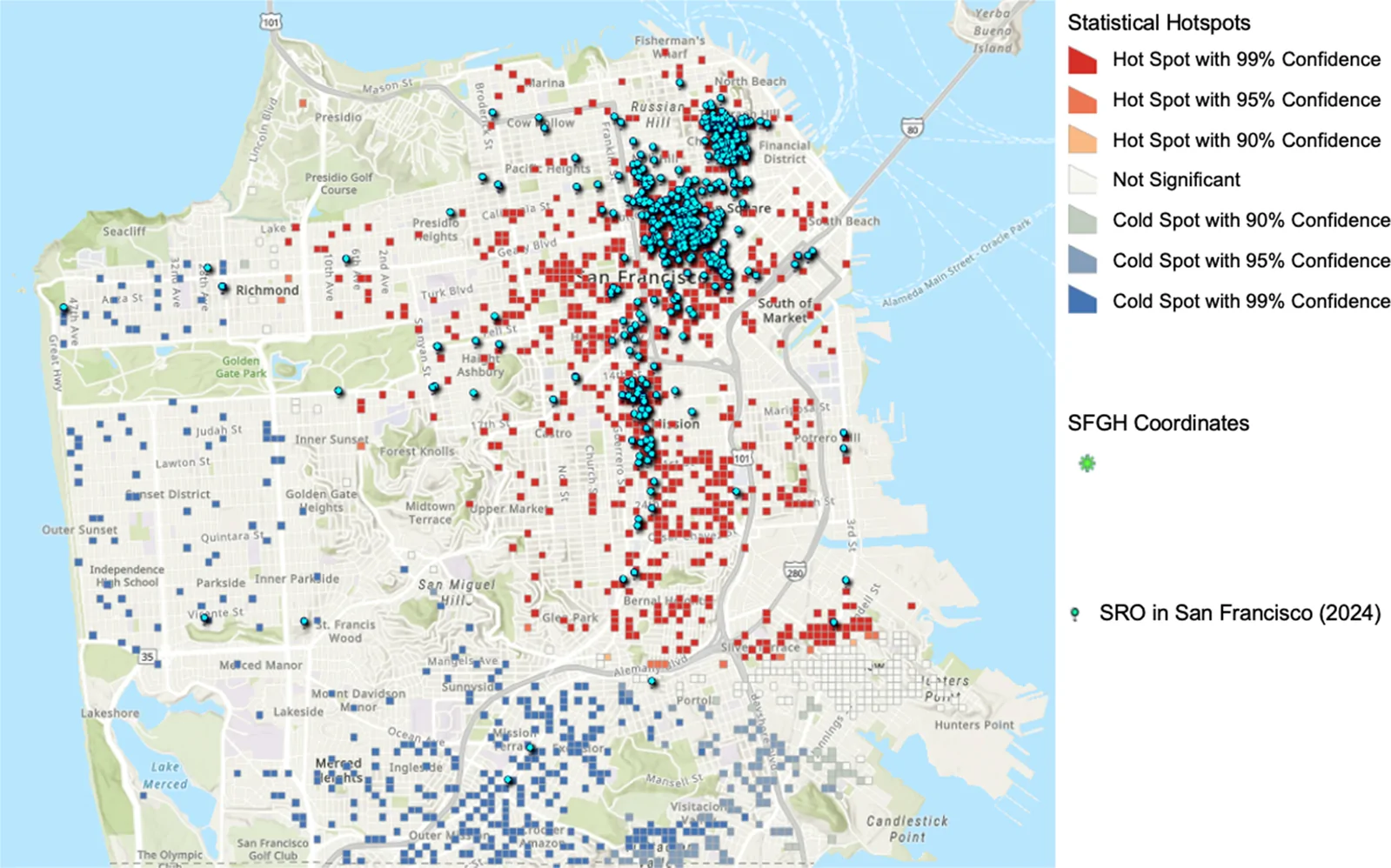
**Statistical Hot Spots of Individuals with Index Heart Failure Hospitalization by Neighborhood Socioeconomic Status (2001-2019) Overlaid with Single Room Occupancy Units in San Francisco (2024)** Statistically significant hotspots of index HF hospitalization overlaid with low-income housing known as single room occupancies (SROs). Most statistical hotspots of index HF hospitalization occur in areas with SROs. Abbreviation as in Figure 5.

When index HF hospitalizations were mapped by geocoded addresses and nSES quintile, lower nSES quintiles were found in San Francisco neighborhoods including the Tenderloin, Mission District, and Bayview/Hunters Point. When statistically significant “hotspots” of index HF hospitalization events were superimposed on neighborhoods grades defined by historic redlining according to the Home Owners’ Loan Corporation, which created a neighborhood ranking system used by local real estate developers and appraisers in over 200 cities in the 1930s,^22^ most statistical hotspots occurred in the lowest grade (D for hazardous) and second-lowest grade (C for “declining”) neighborhoods (Figure 5). When statistical “hotspots” of index HF hospitalization events were superimposed on single residential occupancy unit buildings, or “hotels”, the strongest hotspots coincided with the location of single room occupancy (SRO) buildings (Figure 6). Maps created for “hotspots” of hospital readmission were similar (not shown).

## Discussion

Among people with HF within a municipal health system, the lowest nSES quintile has a 62% higher risk of HF readmission and a 54% higher risk of all-cause readmission compared with the highest nSES quintile. However, nSES is not associated with mortality. Statistical “hotspots” of index HF hospitalization occur in neighborhoods disproportionately affected by redlining policies in the past which restricted access to mortgages particularly for low-income individuals or those identified as racial/ethnic minorities, as well as neighborhoods with high percentages of SRO buildings (Central Illustration). These findings add to prior literature that nSES is associated with HF outcomes but is one of the first studies to investigate both HF and all-cause readmission, as well as mortality, among patients served by a municipal public health system. Furthermore, we extend the concept of “hotspots” of health care utilization, previously used to target high-risk individuals or neighborhoods for specific interventions, to index HF hospitalization events.

**Central Illustration:**
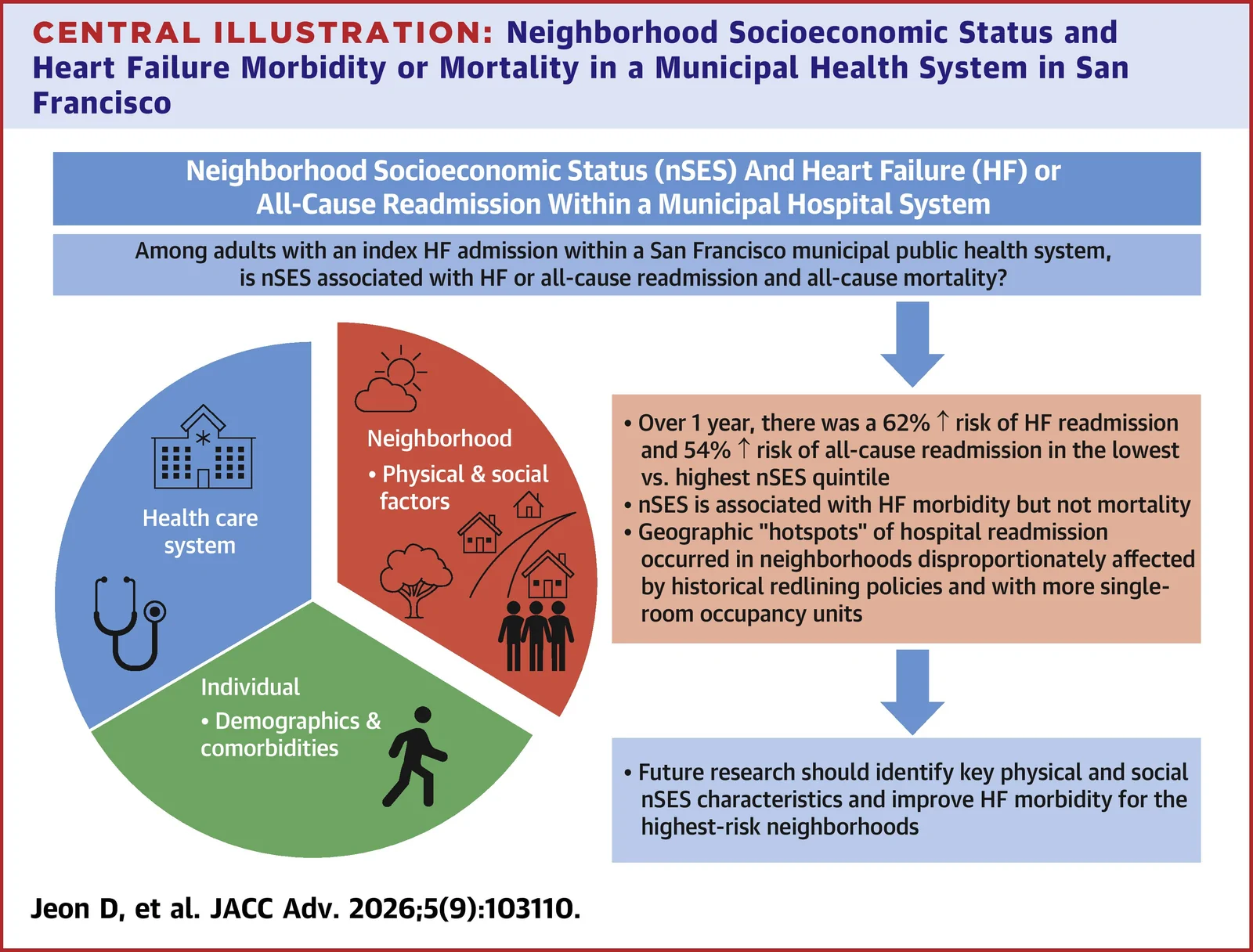
**Neighborhood Socioeconomic Status and Heart Failure Morbidity or Mortality in a Municipal Health System in San Francisco** Neighborhood socioeconomic status is associated with heart failure morbidity, but not mortality, in a retrospective EMR-based cohort study of adults with HF diagnosis and index HF admission within a San Francisco municipal public health system.

Our study expands on prior studies which found that nSES is associated with HF outcomes. Akwo et al^8^ studied Medicare/Medicaid beneficiaries among whom a 1 IQR increase in neighborhood deprivation index was associated with 12% increased risk of incident HF over median follow-up of 5.2 years even with adjustment for individual risk factors including socioeconomic status. Other studies have focused on HF readmission. In a study of adults ages 65 years and older with newly diagnosed HF in a single university health care system, Dhingra et al^23^ found greater neighborhood disadvantage was associated with a higher risk of recurrent admission over the total follow-up period of 5.6 years. Our study adds to the existing literature by investigating the relationship between nSES and both heart-failure or all-cause readmission over 1 year within a municipal health care system.

On the other hand, prior studies have supported different conclusions about the potential association between nSES and mortality. We did not find any significant association between nSES and all-cause mortality. However, Patel et al^24^ found that Black individuals had higher 30-day HF readmission or all-cause mortality than White individuals in all neighborhood social deprivation index quartiles. In a national French study of adults with HF hospitalization in 2018, Guillermo et al^25^ found that the most deprived socioeconomic index quintile had 11% higher rate of mortality compared with the least deprived socioeconomic index quintile.^25^ These were both larger studies, and our null finding between nSES and mortality may be due to limited sample size and follow-up.

In contrast, other studies including Mathews et al^12^ found no association between area deprivation index and mortality specifically in patients with HFrEF. Bikdeli et al^11^ found that nSES is not associated with 6-month mortality, only 6-month readmission, among patients with HF in internal medicine and cardiology practices across the United States. Although these studies again differ in significant ways, including study populations and which individual-level socioeconomic factors were adjusted for, these studies and ours point to the complexity of how nSES may be associated with mortality.

In addition, our study adds to prior studies that have found associations between historic redlining grades assigned to neighborhoods in the United States and adverse health outcomes including cardiometabolic outcomes, injury or violence, and asthma.^22^ Mentias et al^26^ found that residential zip code redlining proportion is associated with an increased risk of HF incidence among Black patients even when controlling for zip code-level socioeconomic deprivation. Here, we find that statistical “hotspots” of index HF hospitalization events are more likely to be found in neighborhoods disproportionately affected by U.S. redlining policies in the 1930s. Although any potential causal mechanisms between historical redlining and adverse health outcomes are complex, and these findings must be interpreted cautiously, our study is an impetus to further investigate how public policy and neighborhood characteristics can shape individual and neighborhood risk factors and ultimately HF outcomes.

Beyond redlining, we also investigated a different neighborhood housing characteristic: the presence of SROs. To our knowledge, our study is the first to examine hotpots of index HF hospitalization events in the context of individuals living in SRO units, or “SROs”. The history of SRO units, housed in buildings called “hotels”, date back to the early 20th century, when many were built to house transient occupants including short-term laborers, low-wage workers, and immigrants. Since then, SROs have become one of the most important and largest affordable housing option for low-income individuals and families in San Francisco, even as pressures of urban development and renewal led to the demolition or conversion of many SROs.^27^ As of 2014, there were approximately 581 SRO buildings in San Francisco, with an estimated 30,000 tenants living in SRO hotels of which 88% are located in 6 zip codes. In a 2016 health impact assessment of SRO hotels in San Francisco, individuals living in these zip codes had up to 2 times higher rate of admission for HF and up to 3 times higher rate of admission for chronic obstructive pulmonary disease compared to other San Franciscans.^27^ Although few other studies have compared health outcomes of individuals living in SROs compared to the rest of San Francisco, one study found that despite making up 3% of the adult population in San Francisco, SRO residents accounted for more than 25% of overdose deaths during the study period.^28^ These data suggest that SRO residents are at disproportionately high risk for adverse health outcomes, including HF admissions. Thus, our study finds that statistically significant “hotspots” of SRO occur in neighborhoods disproportionately affected by redlining in the past and with higher SRO numbers.

Identifying “hotspots” of adverse health care outcomes is an initial step toward designing, implementing, and evaluating programs and policies to improve health outcomes for individuals and the communities in which they reside. Efforts to improve health care outcomes for individuals with high health care utilization led to the Hospital Readmission Reduction Program, a Medicare value-based readmissions program which penalized hospitals with excessive admissions beginning in October 2012. Programs that have targeted high health care utilizers include the Camden Coalition of Healthcare Providers, a multidisciplinary program in New Jersey which aimed to reduce hospital readmissions by supporting patients closely during the postdischarge period. Finkelstein et al^29^ evaluated the program through a randomized controlled trial of 800 hospitalized patients with medically and socially complex needs and a history of hospitalization within the past 6 months, where the treatment arm received intensive clinical and social postdischarge services from a multidisciplinary team including community health workers, nurses, and social workers compared with a control arm who received usual postdischarge care. In contrast to prior studies of high health care utilizers, Finkelstein et al. found no difference in the primary outcome of readmission within 180 days after hospital discharge, nor the secondary outcomes of number of readmissions, hospital days, charges, or mortality 180 days after discharge associated between the treatment and control groups. Although no similar large-scale randomized controlled trials have been performed of intensive individual multidisciplinary interventions aimed at reducing hospital readmission, a subsequent secondary analysis of the same randomized controlled trial by Finkelstein et al. found that hospital readmissions were lower among patients with higher participation in the intervention group, suggesting that higher adherence to the treatment was a key mediator.^30^ Further research is needed to investigate how to improve treatment and health care outcomes for individuals with high health care utilization.

In contrast, other studies of community-based programs have been shown to be effective. Models of community-based care, such as bringing in ancillary health care providers into nontraditional settings like barbershops to help identify and manage hypertension,^31^ or bringing community health workers into HF management,^32^ have been shown to be cost-effective while reducing morbidity and mortality associated with chronic cardiovascular conditions. Such programs could ideally serve neighborhoods at highest risk of HF hospitalization and readmission. For example, identifying neighborhoods with high percentages of SRO residents may help target community-based interventions, including training SRO management staff or incorporating multidisciplinary clinical teams to provide care directly to residents of SROs. Similarly, 1 study found that healthy lifestyles mediated only part of the association between social vulnerability and index HF, suggesting the need to again focused on structured programs and policies at the community and neighborhood level. Targeting programs and policies to reduce HF morbidity and mortality will likely require multidisciplinary efforts to build, measure, and scale interventions with community input using health services and implementation sciences to target systemic determinants of health, including neighborhood physical and social environment, as well as individual risk factors and behavior.

### Study Limitations

We also note specific limitations of this study including the lack of individual-level socioeconomic data. We cannot extrapolate findings about people living in specific neighborhoods to every individual living in these areas. We excluded patients without geocoded address, such as individuals experiencing homelessness, unstable or marginal housing, or living in temporary residences. Given hospital readmission data are not available for patients without a residential address, we cannot exclude the possibility that patients with residential address differ in their risk of HF or all-cause readmission or mortality from those without residential address who are likely at highest risk of adverse health outcomes^33^; thus, their exclusion may underestimate the association between nSES and HF morbidity and mortality. Moreover, patients with index HF admission had a higher burden of specific comorbidities and substance use than those without index HF admission, which limits the external validity of our findings to the rest of the patient population within this municipal public health care system or more broadly to the U.S. population with HF.

In addition, over the study period, there is no specific time associated with when people lived at the residential addresses listed, and individuals could have different lengths of time between when they lived at an address and the time of HF hospitalization, readmission, or death. However, prior data have shown that most people, especially if living in lower nSES areas, do not generally move nSES quintiles even if moving physical addresses.^34^ Separately, gentrification in San Francisco has affected the physical and social characteristics of neighborhoods over time while causing involuntary displacement of lower-income individuals.^35^ Although the question of how gentrification may affect neighborhood characteristics and health outcomes over time is complex and not captured in this study, growing evidence suggest that certain populations, particularly individuals who identify as racial/ethnic minorities or have lower socioeconomic status, are more likely to have negative health outcomes due to gentrification.^36^ Furthermore, we may not have captured all readmissions for our patients due to hospitalization events outside of our health care system, and those who live in higher nSES neighborhoods may be more likely to seek care at health facilities located in their neighborhoods.

In conclusion, our study is a large, well-characterized EMR-based retrospective cohort study of individuals with HF within a municipal hospital system that finds significant associations between nSES and HF morbidity within a municipal hospital system. Ultimately, identifying highest-risk neighborhoods for HF or all-cause readmission is 1 initial step toward addressing these issues. Further research should investigate which specific physical and social attributes of nSES are associated with HF and all-cause readmission or mortality, as well as the measurement and implementation of community-level interventions and public health policy measures to reduce index HF hospitalization, readmission, and morbidity.

Perspectives **COMPETENCY IN MEDICAL KNOWLEDGE:** Beyond individual-level clinical and demographic factors, lower neighborhood-level socioeconomic status is associated with increased HF or all-cause readmission, but not mortality. Specific neighborhoods are at the highest risk for HF or all-cause readmission.

**TRANSLATIONAL OUTLOOK:** Identifying aspects of neighborhood health contribute to HF outcomes is important to guide targeted public health interventions for “hotspot” neighborhoods as well as future health outcomes, policy, and implementation science research.

## Funding support and author disclosures

Dr Durstenfeld is funded by 10.13039/100000002National Institutes of Health/10.13039/100000050National Heart, Lung, and Blood Institute grant K23HL172699. Dr Hsue is funded by 10.13039/100000002National Institutes of Health/10.13039/100000060National Institute of Allergy and Infectious Diseases grant 2K24AI112393. This study was funded by internal funds from the Division of Cardiology at Zuckerberg San Francisco General and was supported by the 10.13039/100006108National Center for Advancing Translational Sciences, 10.13039/100000002National Institutes of Health, through 10.13039/100005595University of California, San Francisco Clinical & Translational Science Institute grant number UL1TR001872. All other authors have reported that they have no relationships relevant to the contents of this paper to disclose.
