# Supplementary material for: Neighborhood Socioeconomic Status and Heart Failure or All-Cause Readmission Within a Municipal Hospital System
Source: JACC Adv. 2026 Aug 19;5(9):103110. doi: 10.1016/j.jacadv.2026.103110 (PMC13520739; doi:10.1016/j.jacadv.2026.103110)
Supplement: Supplementary file 1 [file mmc1.docx]

**Supplemental Table 1:** Multivariable Model for Heart Failure or All-Cause Readmission Over 1 Year After Index Heart Failure Hospitalization

| **nSES Quintile** | **Heart Failure Readmission Over 1 Year^a^** | **All-Cause Readmission Over 1 Year^b^** |
| --- | --- | --- |
|  | **Hazard Ratio (95% Confidence Interval)** | |
| **nSES Quintile 1 (Lowest)** | 1.59 (1.07, 2.37), p=0.023 | 1.52 (1.15, 2), p=0.003 |
| **nSES Quintile 2** | 1.34 (0.89, 2.02), p=0.16 | 1.54 (1.16, 2.04), p=0.003 |
| **nSES Quintile 3** | 1.08 (0.68, 1.7), p=0.75 | 1.18 (0.86, 1.61), p=0.3 |
| **nSES Quintile 4** | 1.53 (0.99, 2.36), p=0.056 | 1.55 (1.14, 2.1), p=0.005 |
| **nSES Quintile 5 (Highest)** | Reference | Reference |

Legend: Multivariable analyses of the association between nSES quintile and HF or all-cause readmission over 1 year after index HF hospitalization adjusted for demographics (age, sex, and self-reported race), comorbidities (hypertension, diabetes, chronic kidney disease, and atrial fibrillation), type of HF (HFrEF), substance use (alcohol, tobacco, methamphetamine, or cocaine), any prescription of any guideline-directed medical therapy medication during study period, outpatient follow-up over 30 days, and insurance type. Hazard ratios compare each nSES quintile with the highest nSES quintile, 5.

^a^Adjusted hazard ratio for heart failure readmission over 1-year after index HF hospitalization: 0.92 (95% CI 0.86-0.99), p-trend=0.027.

^b^Adjusted hazard ratio for all-cause readmission over 1-year after index HF hospitalization: 0.94 (95% CI 0.90-0.99), p-trend=0.015.

Abbreviations: HF = heart failure; HFrEF = heart failure with reduced ejection fraction; nSES = neighborhood socioeconomic status

**Supplemental Table 2:** Multivariable Model for All-Cause Mortality

| **nSES Quintile** | **Survival Over Follow-up Period^a^** |
| --- | --- |
|  | **Hazard Ratio (95% Confidence Interval)** |
| **nSES Quintile 1 (Lowest)** | 0.91 (0.68, 1.21), p=0.500 |
| **nSES Quintile 2** | 0.91 (0.68, 1.23), p=0.555 |
| **nSES Quintile 3** | 0.85 (0.61, 1.18), p=0.339 |
| **nSES Quintile 4** | 0.84 (0.60, 1.16), p=0.286 |
| **nSES Quintile 5 (Highest)** | Reference |

Legend: Multivariable analyses of the association between nSES quintile and all-cause mortality over study period adjusted for demographics (age, sex, and self-reported race), comorbidities (hypertension, diabetes, chronic kidney disease, and atrial fibrillation), type of HF (HFrEF), substance use (alcohol, tobacco, methamphetamine, or cocaine), any prescription of any guideline-directed medical therapy medication during study period, outpatient follow-up over 30 days, and insurance type. Hazard ratios compare each nSES quintile with the highest nSES quintile, 5.

^a^Adjusted hazard ratio for all-cause mortality: 1.00 (95% CI 0.94-1.05), p-trend=0.950.

Abbreviations: HF = heart failure; HFrEF = heart failure with reduced ejection fraction; nSES = neighborhood socioeconomic status
